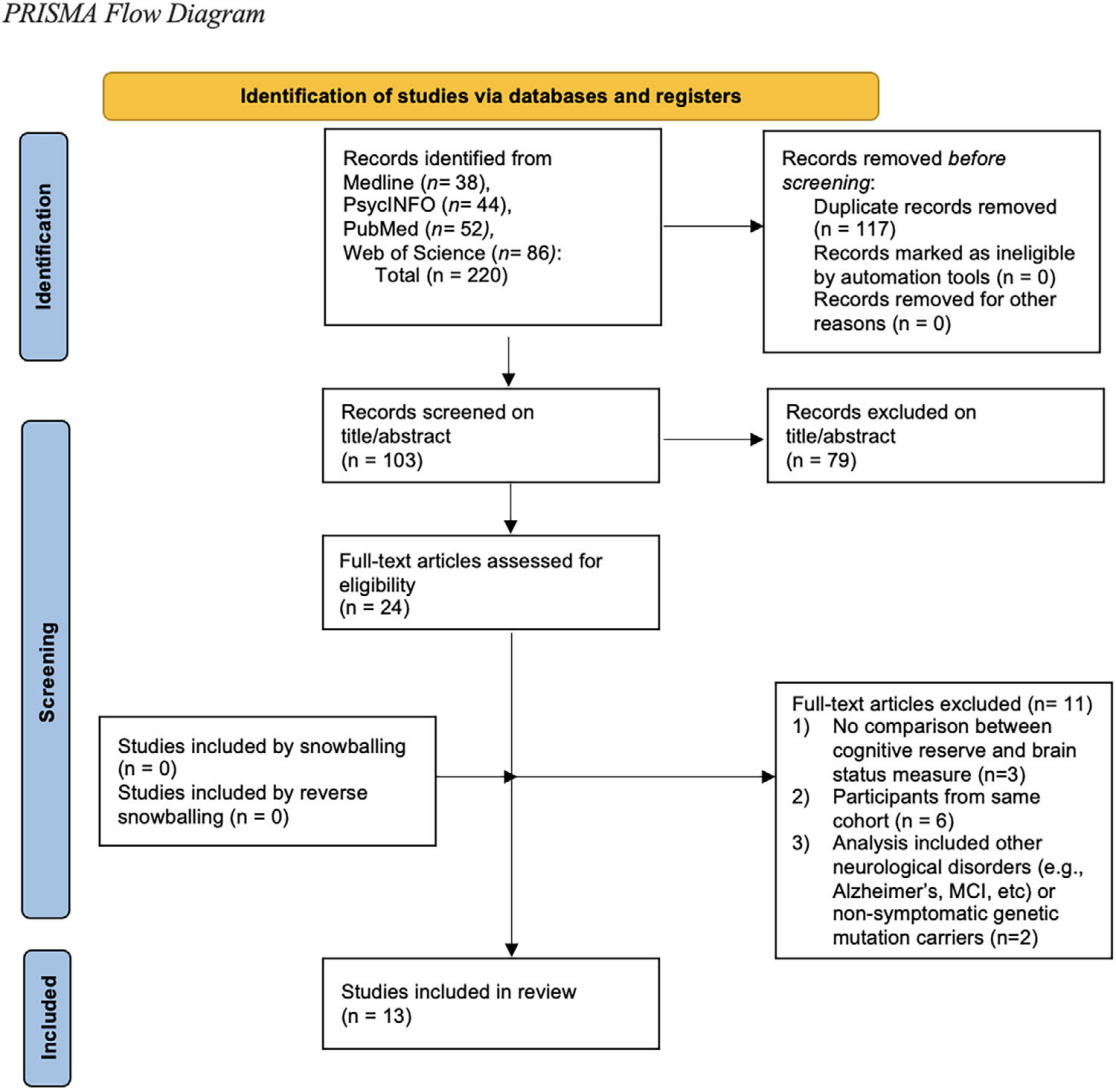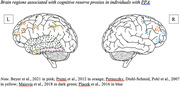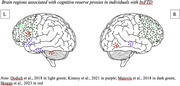# Cognitive Reserve in Individuals with Frontotemporal Dementia: A Systematic Review

**DOI:** 10.1002/alz.085267

**Published:** 2025-01-03

**Authors:** Lauren A Grebe, Jet MJ Vonk, Elizabeth Galletta, Mira Goral

**Affiliations:** ^1^ St. John’s University, Queens, NY USA; ^2^ CUNY Graduate Center, New York, NY USA; ^3^ University of California San Francisco (UCSF), San Francisco, CA USA; ^4^ NYU Grossman School of Medicine, New York, NY USA; ^5^ Lehman College, Bronx, NY USA

## Abstract

**Background:**

In comparison to robust evidence for cognitive reserve (CR) in individuals with Alzheimer’s‐related dementia, the literature on CR in frontotemporal dementia (FTD) is still emerging. A clear consensus on the relationship among CR, brain status, and clinical performance has not been reached. The aims of this systematic review were to: 1) document the FTD disorders represented in this literature and their diagnosis descriptions, 2) classify the sociobehavioral proxies of CR used, 3) identify the tools used to measure disease severity, clinical performance, and brain status, and 4) examine the relationship between CR and brain status in individuals with FTD.

**Method:**

Systematic review of the literature was conducted using a comprehensive range of relevant search terms in Medline, PsychINFO, PubMed, and Web of Science. Eligibility criteria were for studies to: include at least one proxy of CR and one brain status measure for individuals with FTD, be published in a peer‐reviewed journal, and be published in English. The Newcastle‐Ottawa Quality Assessment Scale was used to assess the quality of the included studies and risk of bias based on three domains: participant selection, comparability of included groups, and quality of outcome measures.

**Result:**

A total of 220 titles and abstracts were screened, with 13 studies meeting inclusion criteria. Together, these studies report 1,423 participants diagnosed with FTD. Across studies, three proxies of CR were incorporated as either continuous or categorical variables: education, occupation, and leisure. Seven tools were used to measure disease severity and three neuroimaging tools were used to measure brain status. All included studies reported significant associations between a CR proxy and a brain measure. However, only partial support was demonstrated for the CR theory in individuals with FTD when education, occupation, and leisure involvement were analyzed in relation to disease severity.

**Conclusion:**

The variable results among studies could be related to the different tools used to measure CR, the numerous brain status measures incoporated, and the different ways researchers determine disease severity. Recommendations for future studies include incorporating longitudinal designs, using in‐depth neuropsychological testing, improving measurement of disease duration, and transparant reporting of statistical output.